# High-Performance Work Practices and Interpersonal Relationships: *Laissez-Faire* Leadership as a Risk Factor

**DOI:** 10.3389/fpsyg.2022.854118

**Published:** 2022-04-29

**Authors:** Denise Salin, Elfi Baillien, Guy Notelaers

**Affiliations:** ^1^Department of Management and Organisation, Hanken School of Economics, Helsinki, Finland; ^2^Department of Work and Organization Studies, Faculty of Economics and Business, KU Leuven Campus Brussels, Brussels, Belgium; ^3^Department of Psychosocial Science, Faculty of Psychology, University of Bergen, Bergen, Norway

**Keywords:** incivility, competition, *laissez-faire* leadership, high-performance work practices, moderated mediation

## Abstract

Although high-performance work practices (HPWPs) have been shown to increase organizational performance and improve employee attitudes, it still remains unclear how they impact interpersonal relations in the workplace. While some argue that HPWPs lead to better interpersonal relations, others fear that HPWPs may increase competition and uncivil and abusive behaviors. In response to this, our aim is to examine whether and when HPWPs are associated with increased levels of competition and thereby more incivility. Given recent interest in how HR practices and leadership may interact to produce certain outcomes, we study *laissez-faire* leadership as a possible moderator. A survey was conducted in Belgium (*n* = 374), and a mediated moderation analysis using SEM performed using Mplus. The results suggest that in the absence of *laissez-faire* leadership, HPWPs are associated with less incivility, thus suggesting better interpersonal relations. However, the results also show that HPWPs may lead to increased competition and thereby somewhat more incivility, under conditions of *laissez-faire* leadership. The results thus point to the importance of studying interactions between HR practices and leadership in trying to understand employee outcomes. In terms of practical implications, the results suggest that investing in HPWPs may reduce incivility and thereby improve relationship wellbeing. However, HPWPs need to be combined with active leadership to avoid undesirable negative consequences.

## Introduction

During the past decades, there has been growing interest in how human resource practices, in particular high-performance work practices (HPWPs), affect not only organizational performance, but also employee wellbeing (Guest, 2017). Discussions of HPWPs practices and wellbeing have been permeated by the debate around mutual gains versus conflicting outcomes (Kroon et al., 2009; Peccei and Van De Voorde, 2019). In other words, it has been debated whether HPWPs practices benefit both organizations and individual employees, or whether HPWPs practices benefit organizations at the expense of employee wellbeing. So far studies have predominantly focused on happiness wellbeing, such as commitment and satisfaction, and health, such as stress and burnout (Van De Voorde et al., 2011; Peccei and Van De Voorde, 2019). However, conceptualizations of workplace wellbeing typically include three different forms of wellbeing, encompassing relationship wellbeing in addition to happiness and health (Grant et al., 2007; Van De Voorde et al., 2011). Still, few studies have looked at the relationship between HPWPs and relationship wellbeing and even fewer at the boundary conditions that affect the association between HPWPs and relationship wellbeing. Our study seeks to address these gaps.

High-performance work practices refer to progressive human resource practices designed to increase employee skills and degrees of effort (Takeuchi et al., 2007). HPWPs have garnered extensive attention, as they have been shown to enhance employee motivation and performance, and seemingly contribute to an organization’s competitive advantage (Appelbaum et al., 2000; Combs et al., 2006; Wood, 2021). Studies have shown that HPWPs have numerous positive outcomes for employees, including increased commitment, satisfaction, and psychological empowerment (Messersmith et al., 2011; Hauff et al., 2018; Peccei and Van De Voorde, 2019), which may in turn explain increases in performance at both the individual and organizational level. However, more critical voices have warned that HPWPs may also increase employee stress and exhaustion (Ramsay et al., 2000; Godard, 2001; Van De Voorde et al., 2011; Zhang et al., 2013). High commitment may also lead to undesirable consequences. For instance, Xu et al. (2020) showed that by increasing commitment and reducing intentions to leave, high-performance work systems also strengthened the relationship between abusive supervision and employee silence. Furthermore, a prevailing fear in some existing research is that HPWPs may damage interpersonal relations and give rise to competition, undermining, and abusive behavior, toward both subordinates and peers (Samnani and Singh, 2014; Ashkanasy et al., 2016; Pichler et al., 2016). Yet, empirical research to support or refute these claims is notably sparse. In a Finnish study on HPWPs and workplace bullying, HPWPs were found to reduce rather than increase the risk of bullying (Salin and Notelaers, 2020). The conflicting findings thus point to the need to increase our understanding of whether HPWPs have positive or negative outcomes on interpersonal relationships.

It has been hypothesized that contextual factors may determine how HPWPs affect employees, leading to positive results under certain circumstances and negative under others (Han et al., 2020). Empirical research shows some support for this. For instance, employee perceptions of supervisor intent affect employee reactions. Employees may then experience performance management as supportive, when they trust that the supervisors are genuinely interested in their growth and development, but stressful when they believe supervisors act out of mere self-interest (e.g., Russell et al., 2016). Similarly, the nature of the employee–employer relationship has been shown to determine whether HPWPs lead to engagement or exhaustion (Zhang et al., 2013). That is, HPWPs lead to engagement when employees perceive a social exchange relationship, but exhaustion when they perceive merely economic exchange. Moreover, coercive control has been shown to negate the negative relationship between HPWPs and counterproductive workplace behaviors (Shaffer and Darnold, 2020). In line with such examples, there have been calls for more studies on when and under what circumstances employees will experience positive versus negative outcomes of HPWPs (Marler and Fuller, 2016; Wang et al., 2019; Han et al., 2020).

In this study, we focus on competition and the enactment of incivility as possible dark side effects of HPWPs and examine whether *laissez-faire* leadership is a circumstance under which such dark side effects manifest. We chose incivility as a variable of interest, because this form of employee mistreatment captures also low-intensity and milder forms of mistreatment, such as rudeness (Andersson and Pearson, 1999). Incivility is more common than more severe acts, such as aggression or bullying. We wanted to focus on a form of mistreatment that occurs (also) among peers, not merely downwards. Competition was chosen as the mediator since this is a mechanism that has typically been argued to link HPWPs with poorer social relations and incivility (Spector, 2016). *Laissez-faire* leadership, in turn, was chosen because of claims that HRM and leadership need to be aligned if we want to reap the benefits HRM may offer (Leroy et al., 2018). *Laissez-faire* has been argued to be a destructive form of leadership, associated with poorer employee attitudes, decreased wellbeing, and more interpersonal problems and mistreatment (Judge and Piccolo, 2004; Hauge et al., 2007; Skogstad et al., 2007; Harold and Holtz, 2015). Given the lack of previous research on our topic, we chose to seek a heterogenous sample, rather than focusing on any specific sector. Our cross-sectional study is a first attempt to shed more light on how HPWPs and *laissez-faire* leadership may interact to possibly affect incivility through increased competition.

Our contribution to the literature on high performance is two-fold. First, we contribute to insights into the dark side of HPWPs, showing that when combined with *laissez-faire* leadership, HPWPs may indeed lead to increased competition and thereby incivility among colleagues. Yet, our results also suggest that, generally, HPWPs reduce the risk of incivility. Rather than clearly supporting either a mutual gains perspective or critical perspective on HPWPs, the results suggest that the outcomes may be dependent on contextual factors. Secondly, we contribute to the literature on HRM by pointing to interactive effects between leadership and HRM, an issue predominantly overlooked by the research to date, as the two streams have largely been studied in isolation from each other (Leroy et al., 2018). In particular, we show that poor leadership may undermine the effects of HPWPs by giving rise to negative consequences.

## High-Performance Work Practices and Interpersonal Relationships

### High-Performance Work Practices and Possible Negative Effects

As discussed in the introduction, there has been a longstanding debate on whether HPWPs lead to positive outcomes for both organizations and employees or better organizational performance at the expense of the employees. This has also been referred to as the mutual gains versus conflicting outcomes debate (e.g., Kroon et al., 2009; Peccei and Van De Voorde, 2019). Although results have been mixed, recent reviews suggest there is more support for the mutual gains perspective (Van De Voorde et al., 2011; Peccei and Van De Voorde, 2019); in particular, that HPWPs appear to have clearly positive effects on employee happiness, such as engagement and job satisfaction. However, there is still only limited evidence regarding effects on relationship wellbeing (Van De Voorde et al., 2011).

Some researchers have argued that the line between motivation and abuse may at times be very thin (Ashkanasy et al., 2016), and that by increasing job stress and frustration, HPWPs may create fertile ground for abusive supervision and rude/abusive behavior among colleagues (Samnani and Singh, 2014; Pichler et al., 2016). This, in turn, has been argued to give rise to workplace incivility, defined as “low-intensity deviant behavior with ambiguous intent to harm the target, in violation of workplace norms for mutual respect” (Andersson and Pearson, 1999, p. 457). It is thus a form of low-intensity mistreatment in the workplace, including, for instance, rude and disrespectful behavior. Further, it is closely related to other forms of workplace mistreatment, including bullying, abusive supervision, and social undermining, with mostly the low-intensity and ambiguous intent distinguishing workplace incivility from these other concepts (Hershcovis, 2011).

With respect to HPWPs, in particular performance-enhancing compensation systems and performance-based pay have been hypothesized to increase interpersonal mistreatment and deviant behaviors. This builds on the assumption that performance-based remuneration induces social comparison and competition, thereby stimulating deviant behavior, such as bullying (Samnani and Singh, 2014; Gläser et al., 2017). High-performance expectations may make employees perceive colleagues as threats to their own position. Social comparison research suggests that competitive behavior may also manifest in harmful actions, triggering employees to aggressively retaliate against perceived threats (Gläser et al., 2017). This is in line with findings by Guerci et al. (2019), showing a negative relationship between incentive-based pay based on individual performance and relationship wellbeing, measured as whether employees feel they are treated fairly and get along well with their colleagues.

However, not only incentive-based pay, but also HPWPs and high-performance climates more generally have been feared to increase negative interpersonal behavior and enhance competitiveness among colleagues (Spector, 2016). This is because the performance standards, reflected not only in compensation and performance appraisal practices, but in recruitment and training alike, typically emphasize excellence and performing to an extraordinary level, whereas merely doing the job correctly and meeting deadlines is considered insufficient. All these processes may thus increase social comparison, which may make colleagues appear as threats and competitors. Competition in turn may lead to undermining (Duffy et al., 2002), aggression and counterproductive behavior toward teammates (Spector, 2016), and workplace bullying (Salin, 2003). Thus, it is possible that competitiveness is a mechanism through which HPWPs may stimulate more incivility and other forms of mistreatment in the workplace.

Nonetheless, empirical research on the effects of HPWPs is far from consistent in linking HPWPs to more negative interpersonal relationships. In fact, in their review of the literature, Van De Voorde et al. (2011) found that HPWPs were typically associated with improved, rather than decreased, relationship wellbeing. As such, the debate on the association between HPWPs and interpersonal relationships is characterized by opposing views and arguments. This opens up the possibility that whether HPWPs have positive or negative effects on interpersonal relationships may depend upon contextual factors. In the next section, we turn to one such possible moderating factor, the leadership style of the closest supervisor.

### Interactions Between HRM and Leadership

Although the HRM and leadership research streams are in essence both about how to effectively manage people in organizations, it has been highlighted that the two fields remain largely separate and have been studied in isolation from each other (Leroy et al., 2018). In other words, we know surprisingly little about the relationship between HRM and leadership and how they interact to shape employee behavior. Calls have therefore been made for more research studying how leadership and HR interact (Leroy et al., 2018).

In research on HPWPs, issues around leadership have come to the fore, primarily through highlighting the role of line manager implementation of the practices. It has been argued that the line manager implementation is key to understanding employee perceptions of HPWPs (Sikora et al., 2015), which in turn largely determines employee reactions to them (Bowen and Ostroff, 2004). Bowen and Ostroff (2004) highlight the role of a visible supervisor in combination with a strong HR system as a critical element in the HRM–performance relationship.

Although leadership has to date mostly been linked to the actual implementation of HPWPs, leadership and HR may also interact in other ways to co-determine employee behaviors (Leroy et al., 2018). Within a supplementary fit framework, Leroy et al. (2018) discuss the need for leaders and HR to be aligned, in their underlying values and the messages they send, for effects to be optimal. Without a fit between HR and leadership, organizations are unlikely to reap the benefits HR may otherwise offer. In line with this, Hai et al. (2020) found that transformational leadership strengthened the effect of HPWPs on employee engagement and organizational citizenship behaviors. However, if active and constructive leadership can be assumed to strengthen the HR system, and increase the likelihood of desired outcomes, it is also possible that a lack of such leadership may weaken the HR system, and even give rise to undesired consequences. It has been argued that should HRM and leadership contradict each other, followers may even become confused about what is expected of them (McClean and Collins, 2019). As a result, HRM and leadership may undermine each other’s efforts.

In this paper, we thus examine the potentially negative role of passive leadership behaviors, by examining how *laissez-faire* leadership may interact with HPWPs to increase the risk of undesired outcomes. *Laissez-faire* leadership has been defined as an abdication of the responsibilities and duties assigned to the superior, in essence describing an absence of leadership (Judge and Piccolo, 2004; Skogstad et al., 2007). Essentially, a *laissez-faire* leader demonstrates little involvement in the work, providing minimal guidance and support, and typically hesitating to take action, for instance by avoiding involvement in problem-solving and conflict management.

*Laissez-faire* leadership has been associated with both poorer employee attitudes (Judge and Piccolo, 2004), and decreased wellbeing (Skogstad et al., 2007; Usman et al., 2020). Moreover, *laissez-faire* leadership has been found to be an antecedent of interpersonal problems, such as conflicts, incivility, and other forms of mistreatment (Hauge et al., 2007; Skogstad et al., 2007; Harold and Holtz, 2015), whereas constructive leadership has been found to reduce the risk of interpersonal mistreatment, such as bullying (Cooper-Thomas et al., 2013). In a Norwegian study, Ågotnes et al. (2018) found that *laissez-faire* leadership not only increased the risk of bullying behavior in its own right, but also strengthened the relationship between co-worker conflicts and bullying. In other words, it played a vital role in how and when co-worker conflicts developed into systematic mistreatment. In a similar way, *laissez-faire* leadership may interact with other organizational factors in producing undesirable outcomes and reducing positive employee outcomes.

**Figure 1:**
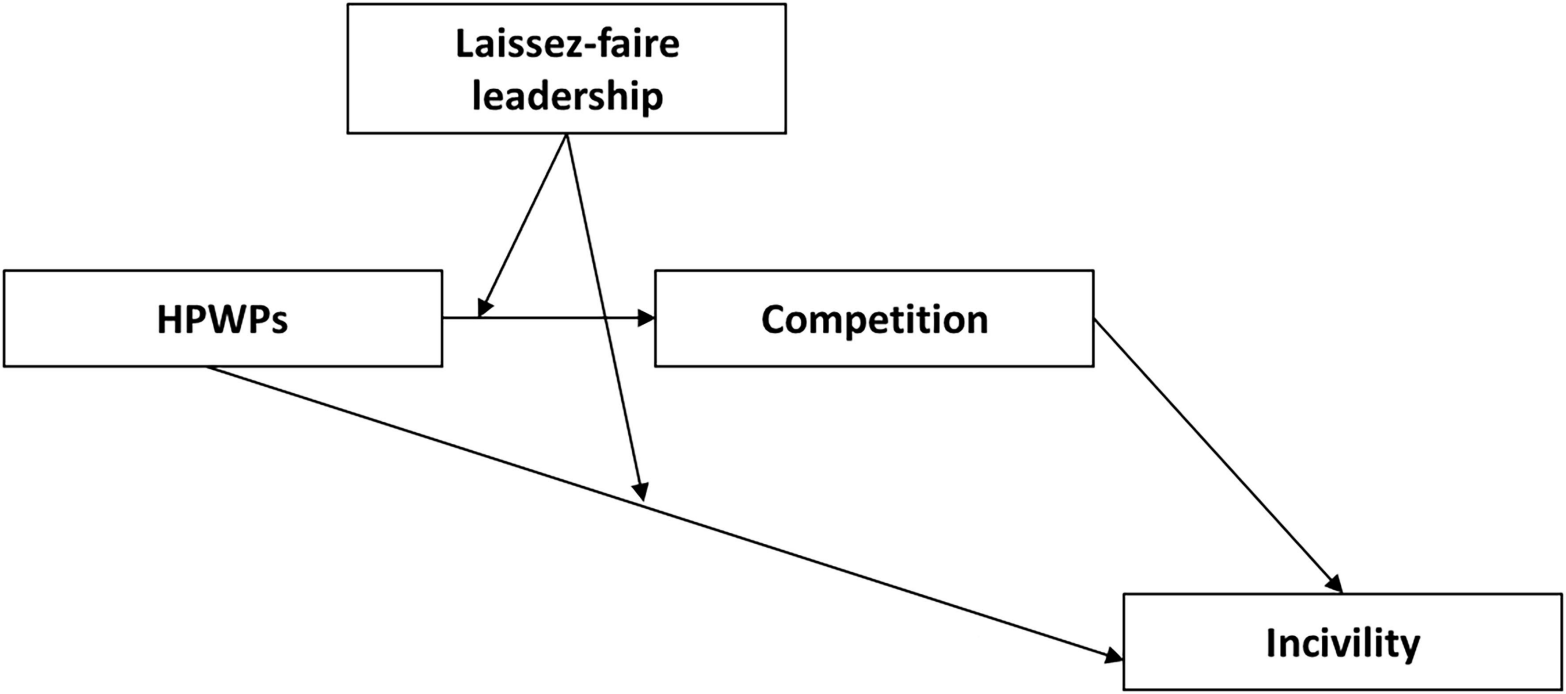
Conceptual model.

Despite the many positive outcomes associated with HPWPs, these practices have also been reported to potentially lead to work intensification and increased demands (Ramsay et al., 2000; Van De Voorde et al., 2011; Zhang et al., 2013; Han et al., 2020). From a stress theoretical perspective (e.g., JD-R, JDCS; Bakker and Demerouti, 2007), it is therefore important that employees also have the resources and the support needed to offset the potentially negative effects stemming from such increased demands. Findings on servant leadership and HPWPs suggest that such leadership may buffer some of the strain associated with HPWPs (Wang et al., 2019). This points to the importance of an active leader who provides guidance and support, while a *laissez-faire* leader most likely would not. Therefore, it is more likely that negative effects of HPWPs would surface under passive leadership. Without a leader who is actively involved, and actively engages in problem-solving and conflict management, it is likely that, for instance, performance management may result in negative competition and incivility between peers. Similarly, the lack of clarity and guidance under high stress may result in negative interpersonal behavior, in particular when the leader does not intervene at an early stage. We argue that it is more likely that negative effects of HPWPs surface under *laissez-faire* leadership and hypothesize that (Figure 1):

*H1*: When *laissez-faire* leadership is high, HPWPs are associated with more incivility, through increased competition.

## Materials and Methods

### Sample

An online survey was conducted in 2018 in Belgium among Flemish-speaking employees in a variety of sectors as to obtain a heterogeneous sample of participants to our study, ensuring variance in our study variables. The survey was carried out in Flemish, and back translation was used to ensure high-quality translations. The respondents (*n* = 374) worked in retail (10.5%), healthcare (16.2%), transportation (8%), finance (17.7%), public services (8.9%), education (14.3%), consultancy (13.6%), and government (10.8%). They were recruited in two ways. First, 16 organizations, selected from a list of organizations belonging to a range of sectors part of the faculty’s internship program as well as through the research group’s professional network, agreed to participate by either contacting the employees of a certain department (12 organizations)—this was the case when other studies had already taken place in several departments and management chose to avoid over-questioning their staff—or randomly selecting a sample of employees from several establishments of the organization as to enlarge variance in responses (4 organizations). A contact person in the HRM department facilitated our access to the employees. Participation was encouraged by underscoring that employees could take part voluntary and that the data gathered were confidential and for scientific research purposes only. The response rate was high, 66.9% of those contacted responded to the questionnaire. A total of 333 responses were obtained this way. Second, snowballing was applied through e-mail and social media, which led to a further 41 participants. The sample’s mean age reached 38 years (SD = 12). Of the respondents, 77% were female, 76% had a full-time contract, and 21% held a supervisory position. The sample participants in were highly educated, with 76% having obtained at least a bachelor’s degree.

### Measures

High-performance work practices (HPWPs) were measured with 24 items, taken from Chuang and Liao (2010). The items measured practices related to six different areas of HR: staffing (e.g., “Recruitment emphasizes traits and abilities required to perform well in this organization”); training (e.g., “My organization invests considerable time and money in training”); performance appraisal (e.g., “Performance appraisals are based on objective, quantifiable results”); compensation (“Employee salaries and rewards are determined by their performance”); participation (e.g., “If a decision made might affect employees, the organization asks them for opinions in advance”); and, caring (e.g., “My organization has formal grievance procedures to take care of employee complaints and appeals”). Different employees in the same organization may have different experiences of HPWPs, since practices may be implemented differently across employees and employees may differ in their interpretations of these practices. Since the subjective experience of HPWPs has been shown to be a better predictor of employee attitudes and behavior (Liao et al., 2009) we have chosen to focus on employee perceived HPWPs. Replies were given on a five-point Likert scale, ranging from 1 = strongly disagree to 5 = strongly agree. Cronbach’s alpha was 0.86.

Competition was measured with four items (Fletcher and Nusbaum, 2010). Sample items included “My coworkers are highly competitive individuals” and “My coworkers work hard to outperform each other.” Replies were given on a five-point Likert scale, ranging from 1 = strongly disagree to 5 = strongly agree. Cronbach’s alpha was 0.91.

*Laissez-faire* leadership was measured with seven items. Sample items included “My supervisor has a strong tendency to put off making decisions” and “My supervisor leaves subordinates to their own devices.” Replies were given on a five-point Likert scale, ranging from 1 = strongly disagree to 5 = strongly agree. Cronbach’s alpha was 0.92.

Enactment of incivility was measured with the Flemish version of the Short Negative Acts Questionnaire (Notelaers et al., 2019), encompassing nine items. Typically phrased to capture exposure to different negative social acts, this study rephrased the items to capture instead the enactment of these acts (Baillien et al., 2011). Overall, the reported mean scores for the scale were relatively low, most accurately described as incivility based on the validation study by Einarsen et al. (2009). Sample items included “Held back information needed by someone else,” “Insulted someone,” and “Excluded someone from group activities.” Respondents were asked to indicate how often they had engaged in the listed acts, based on the following scale: 1 = never; 2 = now and then; 3 = monthly; 4 = weekly; 5 = daily. Cronbach’s alpha was 0.67.

Control variables. The following control variables were entered in the analyses: age, gender (female = 1), and whether a person manages others (yes = 1).

### Procedures

Before testing our research hypothesis, we discerned whether the measurement model that differentiated a second-order factor structure of HPWPs from competition, *laissez-faire* leadership, and workplace incivility fitted the data.

Due to the limited number of observations, we tested our hypotheses with the scale scores rather than the latent variable model. We also took into account that incivility is measured by frequency indicators (never, sometimes, monthly, weekly, daily) that follow an inverted gamma distribution as opposed to a normal distribution. To accommodate this, we estimated a latent class model in Latent Gold 5.1 (Vermunt and Magidson, 2016). More specifically, we estimated a latent class factor model (Vermunt and Magidson, 2005), which may be defined as an ordered latent class cluster model because the latent classes are ordered in function of frequency of incivility. According to the Bayesian information criterion, a latent class factor model with five levels or latent classes fitted the data best. Once the latent classes of incivility were estimated, we exported the latent classes and used this *a posteriori* classification as the dependent variable (incivility) in our conditional process analysis (see for a similar approach: De Cuyper et al., 2008).

Following Hayes (2013, p. 381–389) with respect to moderated mediation and mediated moderation, we conducted a conditional process analysis using SEM to test our research hypothesis. We applied the Mplus syntax by Stride et al. (2015) to implement Hayes’ (2013) process scripts for moderated mediation. Thereby, we ran 5,000 bootstraps to obtain the parameter estimates of our conditional process model. For the investigation of the interaction effect, we inspected the unstandardized regression coefficients or slopes at three levels of the moderator, that is –1SD, mean (0 SD), and +1SD.

## Results

While testing the measurement model that differentiated a second-order factor structure of HPWPs from competition, *laissez-faire* leadership, and workplace incivility, the ordinal nature of the indicators was accounted for using the WLSVM estimator in Mplus 8.4 (Muthén and Muthén, 2018). The *χ*^2^ of this model was 1,530 with 890 degrees of freedom. Hence, the imposed measurement model did not fit the data exactly (Jöreskog and Sörbom, 1993). Yet, RMSEA was 0.041 and the probability that RMSEA was under the threshold of 0.05 was 1. Thus, the confirmatory measurement model fitted the data approximately. In addition, the descriptive statistics, that is CFI and TLI, were higher than 0.95 (CFI = 0.968; TLI = 0.966). Because the RMSEA was below 0.05 and the descriptive fit statistics were above 0.95 we concluded that the measurement model fitted the data well. The Harman test to check for CMV showed that a single factor model has a very poor fit. Its *χ*^2^ was 9370.151. This represents a very large deterioration of statistical fit. Also, the test where all indicators equally load on CMV factor that is unrelated to the other factors in the model did not lead to an improvement of fit. With one degree of freedom difference the *χ*^2^ decreased only with 0.3, which is not statistically significant.

Table 1 presents the means, standard deviations, and inter-scale correlations for all variables. The correlations table shows that age was not significantly related to any of the study variables. Furthermore, neither gender nor age was significantly related to incivility.

**Table 1:** Descriptive statistics.

	Mean (SD)	1	2	3	4	5	6
1. Competition	2.097(0.953)	0.914					
2. Incivility	1.321(0.298)	0.145^*^	0.668				
3. HPWPs	3.117(0.606)	−0.003	−0.187^*^	0.856			
4. *Laissez-faire*	2.061(0.946)	0.066	0.219^**^	−0.504^**^	0.920		
5. Gender	77% (female)	−0.163^**^	−0.015	−0.099	0.024	–	
6. Supervisor position	21%	0.037	0.057	0.189^**^	0.009	−0.179^*^	–
7. Age	38(12)	−0.041	−0.064	0.083	−0.038	−0.027	−0.086

*Legend: diagonal represents Cronbach α. Off-diagonal represent Pearson correlation*.

* *p < 0.0.05,*

** *p < 0.01*.

**Figure 2:**
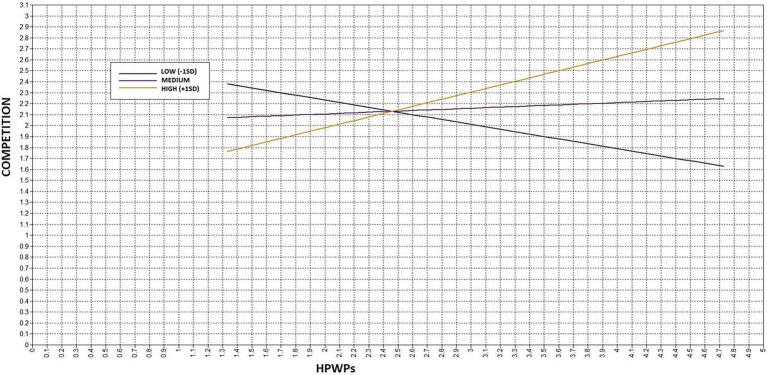
The relationship between HPWPs and competition at three levels of the moderator (*laissez-fair* leadership).

The results of the conditional process analysis support the hypothesis that competition mediates the relationship between HPWPs and incivility under the influence of *laissez-faire* (LF; see Table 2). The simple slopes in Table 3 show that when LF was relatively low (-1SD) or average (0SD), the mediating effect of competition between HPWPs and incivility was not significant. The mediation or indirect effect was only significant—and positive—when LF was relatively high (1SD). Hence, when LF was low or moderate, there was no relationship between HPWPs and incivility *via* competition (see also Figure 2) In other words, competition played no role in the relationship when LF was not high. Yet, when LF was high, there was a relationship between HPWPs and incivility due to competition, and the relationship between HPWPs and competition was significant and positive. It should be noted that +1SD (“high”) corresponds to a value of approximately 3 on the *laissez-faire* scale (*μ* = 2.06; SD = 0.95). This depicts the neutral mid-point of the scale, that is, neither agree nor disagree with the statements.

**Table 2:** Conditional process analysis: standardized regression coefficients after 5,000 bootstraps.

	Competition	Incivility
Gender	−0.160^**^	0.002
Managerial position	0.013	0.091
Age	−0.043	−0.079
HPWPs	−0.314^**^	−0.309^**^
Competition	–	0.119^*^
*Laissez-faire*	−0.667^**^	−0.262^**^
HPWP^*^Laissez-faire	0.672^**^	0.375^*^

* *p < 0.0.05,*

** *p < 0.01.*

**Table 3:** Simple slopes tests after 5,000 bootstraps.

	*Laissez-faire*	Unstandardized effect	BCI low (2.5%)	BCI high (2.5%)
Total effect	Low	−0.531^**^	−0.852	−0.199
	Medium	−0.297^*^	−0.532	−0.061
	High	−0.063^ns^	−0.329	0.198
Indirect effect	Low	−0.039^ns^	−0.115	0.003
	Medium	0.001^ns^	−0.022	0.052
	High	0.041^*^	0.007	0.129
Incivility (direct effect)	Low	−0.471^*^	−0.786	−0.142
	Medium	−0.229^*^	−0.457	−0.001
	High	0.013^ns^	−0.238	0.269
Competition	Low	−0.492^*^	−0.754	−0.109
	Medium	−0.298^*^	−0.464	−0.013
	High	−0.104^ns^	−0.291	0.218

Legend: ns: not significant at the 0.05 level. ^*^: significant at the 0.05 level. ^**^: significant at the 0.01 level. BCI: Bootstrapped confidence interval (5,000 bootstraps).

With respect to the remaining effect between HPWPs and incivility, the results showed that at low and average levels of LF, the relationship between HPWPs and incivility was negative, whereas at high levels of LF there was no significant relationship. Yet again, LF seemed to halt the beneficial effect of HPWPs on incivility, when it was high. Finally, for the sake of rendering the full depth of the results, we assessed the importance of the direct and indirect paths between HPWPs and incivility. Taking into account that the unstandardized total effect of HPWPs was −0.765, and the unstandardized indirect effect (*via* competition) was −0.078, it is clear that LF weighs more on the direct relationship of −0.686 between HPWPs and incivility. The simple slopes clearly indicate that this beneficial effect was nullified under high LF.

## Discussion

While many scholars have argued that HPWPs may have a “dark side” and affect employees negatively (Ramsay et al., 2000; Ashkanasy et al., 2016), empirical research has been inconclusive, some studies suggesting positive outcomes for both employees and employers, and others suggesting negative effects on employees’ wellbeing and interpersonal relationships (*cf.* Van De Voorde et al., 2011). This study was therefore inspired by the need to better understand the circumstances affecting when HPWPs may have detrimental outcomes (e.g., Han et al., 2020). We focus in particular on competition and incivility as undesirable outcomes, as HPWPs have been argued to increase these (e.g., Lewis and Rayner, 2003; Samnani and Singh, 2014; Pichler et al., 2016). In line with our argumentation that negative consequences perhaps manifest only under certain circumstances, we studied the interaction between *laissez-faire* leadership—a style generally associated with negative employee outcomes—and HPWPs, and the resulting impact on incivility. Our results support the assertion that contextual factors matter, as HPWPs were associated with more incivility through competition, but only under conditions of high *laissez-faire* leadership.

In contrast, and perhaps even more importantly, the results suggest that as long as the leader does not rely on a *laissez-faire* leadership style, HPWPs are in fact related to less, rather than more, incivility. While this is at odds with claims typically made in the mistreatment and aggression literature, which tends to portray HPWPs as risk factors for inappropriate treatment (Lewis and Rayner, 2003; Samnani and Singh, 2014; Pichler et al., 2016), it is in line with previous empirical research that suggests HPWPs may reduce workplace bullying (Salin and Notelaers, 2020). Also, the findings are in line with the review by Van De Voorde et al. (2011), who concluded that HPWPs generally impacted employee–employee relationships positively by increasing trust, cooperation, and social exchange. As such, this study questions prevailing notions of HPWPs, which suggest that the practices’ very presence stimulates negative interpersonal behavior and instead suggest they have a protective effect.

Our study points to the importance of contextual factors in predicting under what circumstances HPWPs may have desirable versus undesirable effects on interpersonal behavior. The results suggest HPWPs may be detrimental to interpersonal relations if leaders adopt a *laissez-faire* approach. It thus aligns with other research on HPWPs, that suggests contextual factors may affect whether or not HPWPs negatively affect employees (e.g., Zhang et al., 2013; Russell et al., 2016; Wang et al., 2019; Han et al., 2020). This has implications for the longstanding debate on mutual gains or conflicting outcomes (e.g., Kroon et al., 2009; Van De Voorde et al., 2011). Indeed, the results suggest that rather than inherently leading to positive or negative outcomes for employees, HPWPs may have different effects depending on the circumstances.

In the literature on incivility and bullying, the belief that HPWPs, particularly those linked to performance-based compensation, lead to increased competition among colleagues has often been utilized to explain why HPWPs would negatively affect interpersonal relationships, and in the long run increase incivility and bullying (Samnani and Singh, 2014; Spector, 2016). This study provides a somewhat more nuanced picture. In fact, the study fails to demonstrate a clear relationship between HPWPs and competition, a finding also reported by Salin and Notelaers (2020). However, again *laissez-faire* leadership interacts with HPWPs, so that when HPWPs are combined with high *laissez-faire* leadership, we find increased competition. Our results show that while for low and moderate levels of *laissez-faire* there is no association between HPWPs and competition, for high *laissez-faire* such an association exists and competition actually works as a mediator, as suggested by Samnani and Singh (2014). Thus, the assertion that HPWPs automatically lead to greater competition, and therefore more incivility, is not necessarily correct. Rather, this seems to manifest only under certain circumstances, for instance with high *laissez-faire* leadership, as demonstrated in this study.

Recently, arguments have been made for studying possible interaction between leadership and HPWPs, rather than merely studying the two fields in isolation (e.g., Leroy et al., 2018; Hai et al., 2020). Our study highlights the importance of this, and supports the argument that to achieve optimal outcomes, leadership and HR need to be aligned; otherwise, they may at worst even undermine each other’s efforts. As this study measured HPWPs by asking about employee perceptions of actual practices (which would already involve the implementation of such practices), it shows that the role of leadership goes beyond the mere implementation of HPWPs, and that leader involvement in the day-to-day job also affects the final outcome of the practices. Thus, it points to the need to understand how these two aspects interact to produce desired and undesired employee outcomes. It also suggests that active leadership is needed to offset the potentially negative effects of HPWPs.

### Practical Implications

While earlier research has largely focused on identifying the beneficial and potentially detrimental effects of HPWPs, this study provides preliminary insights into mechanisms that may alleviate or offset some of the detrimental consequences. The study draws attention to the need to combine HPWPs with active leadership, that is, the opposite of *laissez-faire* leadership. If an organization relies on HPWPs but the immediate supervisor engages in *laissez-faire* leadership, that is passive leadership, the results of this study suggest HPWPs may lead to incivility through increased competition.

Active leadership may take many different forms. Although our study does not provide precise insights into the leadership activities needed to ensure that negative consequences do not arise, our data point to the importance of a leader who is involved, who is present, and takes responsibility. Based on the classic meta-analysis by Judge and Piccolo (2004) we know that *laissez-faire* has an especially high negative correlation with transformational leadership (*r* = −0.65), the latter involving idealized influence, inspirational motivation, intellectual stimulation, and individualized consideration (Bass and Avolio, 1994). Given the high negative correlation with *laissez-faire*, it would therefore seem logical to assume that transformational leadership would be likely to be an active form leadership that could prevent the negative consequences from arising. However, it is possible that transactional leadership, building on contingent reward and management by exception, could be enough to signal that the leader is involved, present, and takes responsibility.

Overall, the results point to the importance of coupling HPWPs with active leadership to offset potentially undesired side effects. It is also important to note that the negative effects were already manifest under relatively low levels of *laissez-faire* leadership, at the point where respondents neither agreed nor disagreed with the *laissez-faire* items and chose the neutral mid-point. This would depict a neither truly active or passive leadership style. This suggests that truly rather than somewhat active leadership is needed to counterbalance the demands imposed by HPWPs.

### Limitations and Suggestions for Further Research

Our results rely on a cross-sectional study design which comes with certain limitations. Before addressing those, we underline that a cross-sectional design was chosen because this is the first study that addresses whether *laissez-faire* leadership plays a role in if HPWPs lead to incivility through competition. As Spector (2019) notes “it makes sense to start new areas of inquiry with the most efficient methods to provide initial evidence that a research question is deserving of attention” (p. 129). Furthermore, in order to design a longitudinal study truly capable of addressing causality we need knowledge about the time processes take to have an effect, but also knowledge of alternative explanations to rule out alternative possibilities for the reported findings. This field of research has not evolved to such a level of scientific discovery that both considerations can be taken into account when designing a longitudinal study.

This study relied on single-source, self-reported data. Hence, common method bias may be a problem: some of the observed variation may be attributable to the measurement method rather than true variation in the latent constructs (Podsakoff et al., 2003). To study whether common method bias threatens validity, we conducted a single factor test (Harman, 1979), producing a poorer fit than the confirmatory factor model. Also, a more advanced approach where a common method factor is modeled in addition to the proposed measurement model did not result in an improvement of statistical fit. This seems to suggest common method bias does not pose a substantial threat to our findings. Also, it is worth noting that interaction effects cannot be artifacts of common method variance (Siemsen et al., 2010). In fact, interaction terms can be severely deflated through common method variance, potentially underestimating rather than overestimating interactions.

Although we employed control variables to optimize the use of our design (see Spector, 2019 for more details), the study is cross-sectional. Hence, further research is needed in order to address issues of causality. Strictly speaking, we cannot exclude the possibility that incivility precedes competition and HPWPs. Thus, analyzing conditional processes with such data is far from optimal. Research employing multi-wave designs can provide more specific information on the stability and change of the model variables and on cross-lagged (i.e., over time) relationships. Given the results from our study, scholars may be encouraged to replicate its model using a longitudinal design. However, we need to underline that such an endeavor is far from easy. To be able to draw causal conclusions it is among other things necessary to establish that cause and effect are correlated and to ensure that the cause occurs prior to the effect (see: Shadish et al., 2002). Whereas the first can be established with a cross-sectional design, the second cannot. However, one needs to know the timeframe or the wavelength. Just measuring variables at arbitrary occasions is not sufficient to assess whether X has happened before Y (Spector, 2019, p. 128). In both research on mistreatment and research on HRM and organizational behavior, there is little to no knowledge when it comes to the optimal time lag variables for this study. As a result, a time-intensive longitudinal within design is warranted to put our finger on this pressing question (Spector, 2019). The latter is a resource-intensive enterprise for which building a business case entails at least some empirical support—including of a cross-sectional nature.

Another limitation is the relatively small sample size, due to which we analyzed the mean scores of HPWPs, competition, and *laissez-faire* leadership. This is a common strategy, but does not account for measurement error that may result in an underestimation of the true effects. Our sample also included participants who had been recruited through snowballing. However, additional analyses showed that findings did not change when leaving these participants out. Furthermore, we used a relatively new measure of *laissez-faire* leadership, which may hamper comparison with other studies. However, all factor loadings were above 0.75, and the measure had high reliability.

Our measure of HPWPs relied on employee perceptions of implemented HPWPs, a strategy that has been become increasingly common over the past 20 years, as these perceptions may better predict employee- and organizational-level outcomes (Liao et al., 2009; Beijer et al., 2021). However, employee perceptions of HPWPs may still differ from management perceptions of intended HPWPs, pointing to the need to understand both. The results of this study suggested a negative relation between employee perceptions of HPWPs and incivility (given the leader was not engaging in a *laissez-faire* leadership style). However, this does not automatically mean that management-rated intended HPWPs are related to less incivility. For instance, if organizations introduce performance-based reward systems, but employees do not feel evaluations and compensation reflect actual performance, it may have a very different effect on incivility. Given that we use employee perceptions of implemented HPWPs rather than management-rated practices it is not surprising to see a high negative correlation between HPWPs and *laissez-faire* leadership. A passive *laissez-faire* line manager would be likely to invest less time and effort in implementing intended HR practices, resulting in poorer employee evaluations of existing HPWPs. However, that *laissez-faire* leadership still has a moderating effect suggests that effect of *laissez-faire* leadership goes beyond merely poor implementation.

This study suggests that *laissez-faire* leadership may undermine the positive effects of HPWPs, and even lead to negative outcomes. While the results show that leadership style affects whether or not HPWPs result in less incivility, they also raise questions about whether leadership may similarly affect other HPWPs outcomes. For instance, there is a longstanding debate on whether HPWPs improve or reduce employee health (e.g., Kroon et al., 2009: Van De Voorde et al., 2011). This study points to the importance of examining whether leadership may potentially act as a moderator also of the HPWP–health relationship, to attain a more nuanced picture of that relationship, too. Furthermore, the study has been limited to investigating the effects of passive-destructive forms of leadership on the relationship between HPWPs and outcomes. However, active leadership can take many different forms, as further described in the Full Range of Leadership model (Bass and Avolio, 1994; Avolio et al., 1999). Future research should examine how different active leadership styles, such as transformational and transactional (contingent reward and management by exception—active) leadership interact with HPWPs in producing and potentially strengthening certain positive outcomes. Future research should also examine how management by exception—passive differs from laissez-faire leadership to deepen our understanding of how more passive leadership affects the positive effect of HPWP. Furthermore, future research may also seek to examine how democratic leadership versus authoritarian leadership interact with HPWPs in producing favorable or less favorable outcomes.

## Conclusion

Although a typical argument in the extant research has been that HPWPs are likely to produce a competitive and stressful environment conducive to negative uncivil treatment (e.g., Lewis and Rayner, 2003; Samnani and Singh, 2014; Pichler et al., 2016), the scant empirical research has, to date, failed to find support for this assertion (e.g., Salin and Notelaers, 2020). We therefore hypothesized that such negative outcomes may manifest only under specific circumstances, for instance, in combination with *laissez-faire* leadership. Our results support this assumption, showing that under *laissez-faire* leadership, HPWPs are indeed associated with more competition and thereby incivility. However, active leadership buffers such negative effects. In fact, in the absence of laissez-fair leadership HPWPs reduce the risk of incivility. The results therefore point to the importance of studying interactions between HR practices and leadership in trying to understand employee outcomes of HPWPs.

## Data Availability Statement

The raw data supporting the conclusions of this article will be made available by the authors, without undue reservation.

## Ethics Statement

Ethical review and approval were not required for the study on human participants in accordance with the local legislation and institutional requirements. The patients/participants provided their written informed consent to participate in this study.

## Author Contributions

DS was the lead author and took main responsibility for developing the research idea and conceptualization, with input from co-authors and wrote up the first version of the introduction, theoretical framework, and discussion. EB was responsible for data collection and for reporting about the sample. GN was responsible for data analysis and for the sections on procedures, results, and methodological limitations. All authors contributed to the article, commented upon all sections and approved the submitted version.

## Funding

This work was supported by the Academy of Finland under Grant 308843 and by the Research Council of Norway under Grant 250127.

## Conflict of Interest

The authors declare that the research was conducted in the absence of any commercial or financial relationships that could be construed as a potential conflict of interest.

## Publisher’s Note

All claims expressed in this article are solely those of the authors and do not necessarily represent those of their affiliated organizations, or those of the publisher, the editors and the reviewers. Any product that may be evaluated in this article, or claim that may be made by its manufacturer, is not guaranteed or endorsed by the publisher.
